# Genotypic and Phenotypic Methods in the Detection of MDR-TB and Evolution to XDR-TB

**DOI:** 10.3390/antibiotics14070732

**Published:** 2025-07-21

**Authors:** Natalia Zaporojan, Ramona Hodișan, Carmen Pantiș, Andrei Nicolae Csep, Claudiu Zaporojan, Dana Carmen Zaha

**Affiliations:** 1 Doctoral School of Biomedical Sciences, University of Oradea, University Street 1, 410087 Oradea, Romania; panus.natalia@student.uoradea.ro (N.Z.);; 2 Department of Preclinical Disciplines, Faculty of Medicine and Pharmacy, University of Oradea, 1 December Square 10, 410073 Oradea, Romania; 3 Department of Surgical Disciplines, Faculty of Medicine and Pharmacy, University of Oradea, 1 December Square 10, 410073 Oradea, Romania; 4 Department of Psycho-Neurosciences and Recovery, Faculty of Medicine and Pharmacy, University of Oradea, 1 December Square 10, 410073 Oradea, Romania

**Keywords:** tuberculosis, MDR-TB, XDR-TB, drug susceptibility testing, phenotypic testing, genotypic testing, GenoType MTBDR*plus*

## Abstract

**Background**: Accurate and rapid diagnosis of drug-resistant tuberculosis is essential for initiating appropriate treatment and preventing the transmission of these strains. This study compares phenotypic and genotypic methods of drug susceptibility testing for *Mycobacterium tuberculosis (M. tuberculosis)*. **Methods**: Resistance to first-line drugs, as well as resistance to second-line drugs (fluoroquinolones and aminoglycosides), was assessed using the Löwenstein–Jensen medium phenotypic method and the GenoType MTBDR*plus* genotypic method and analyzed. **Results**: The phenotypic resistance rate was 84.85% for INH (*n* = 56), 46.97% for RIF (*n* = 31), 48.48% for STR (*n* = 32), and 30.30% for EMB (*n* = 20). Of the MDR-TB isolates (*n* = 29), 41.37% were resistant to fluoroquinolones (*n* = 12) and 31.03% were resistant to both fluoroquinolones and injectable aminoglycosides, being classified as XDR-TB (*n* = 9). In addition, 22.73% of the MDR-TB isolates were resistant to all four first-line drugs (*n* = 15). The overall concordance between the line probe assay method and phenotypic testing was 94.74% for RIF and 95.16% for INH. Discordances were identified in three cases for RIF and two cases for INH, where isolates were reported as susceptible by GenoType MTBDR*plus*, but phenotypically resistant. **Conclusions**: Genotypic testing using GenoType MTBDR*plus* provides rapid and accurate results, but some cases of phenotypic resistance are not detected by this method. The results highlight the importance of using combined phenotypic and genotypic methods for accurate diagnosis of MDR-TB, as well as the need to integrate genomic sequencing to improve diagnostic accuracy.

## 1. Introduction

While tuberculosis (TB) is one of the oldest known infectious diseases, it remains one of the most prevalent infectious diseases globally and continues to pose a major threat to public health. According to the World Health Organization (WHO) Global Tuberculosis Report 2023, 10.6 million new cases of TB were recorded in 2022, of which approximately 450,000 were classified as multidrug-resistant tuberculosis (MDR-TB) or rifampicin-resistant tuberculosis (RR-TB) [1].

Over the past decade, the incidence of MDR-TB has increased significantly globally. Although the proportion of primary MDR-TB cases decreased from 3.90% in 2015 to 3.10% in 2020, the total number of cases reached 10.8 million in 2023. This sharp increase, which occurred after the COVID-19 pandemic, suggests that, following a period of underreporting during the pandemic, the resumption of diagnostic and monitoring activities, as well as the restoration of resources, led to a much higher number of cases being reported [2].

In the European region, it is estimated that 225,000 people will become ill with TB in 2023, equivalent to an average incidence of 24 cases per 100,000 inhabitants (2.1% of the total global burden of TB) [3]. In 2022, Romania recorded one of the highest TB rates in the European region, with 48 cases per 100,000 inhabitants [4]. A major challenge is MDR-TB, with approximately 350 cases notified annually, according to the National Guideline—Drug-Resistant Tuberculosis Management 2023. Treatment of MDR-TB and extensively drug-resistant tuberculosis (XDR-TB) requires expensive and long-term medication, is often accompanied by significant side effects, and has low success rates (56% for MDR-TB and 39% for XDR-TB), highlighting the need for more efficient diagnosis and tailored therapeutic strategies [5,6,7].

MDR-TB refers to strains of *Mycobacterium tuberculosis (M. tuberculosis)* resistant to at least isoniazid (INH) and rifampicin (RIF), which are considered first-line treatments. To optimize the management of MDR-TB, the WHO classifies these cases into five categories: isoniazid-resistant TB (Hr-TB), rifampicin-resistant TB (RR-TB), multidrug-resistant TB (MDR-TB), pre-extensive drug-resistant TB (pre-XDR-TB), and extensively drug-resistant TB (XDR-TB), each of which requires therapeutic approaches tailored to the complexity of resistance [8,9].

A study published in *Frontiers in Pharmacology* reported that between 2010 and 2019, the global MDR-TB rate declined by an average of 1.36% per year, but in many resource-limited regions, the incidence of MDR-TB remained stable and that of XDR-TB was increasing, highlighting the need for more effective control and treatment strategies to prevent disease transmission, reduce associated mortality, and improve patient outcomes [10,11,12]. In the absence of early detection of drug-resistant tuberculosis (DR-TB), the risk of therapeutic failure increases, favoring the emergence of pre-XDR and XDR-TB forms [13].

In the past, the identification of TB resistance in the laboratory relied on phenotypic methods, which involved growing *M. tuberculosis* in the presence of antibiotics, known as phenotypic drug susceptibility tests (DST). Today, diagnosis has shifted to molecular techniques that detect mutations in genes involved in resistance, called molecular DST [14].

Among the conventional phenotypic methods approved for drug susceptibility testing of *M. tuberculosis*, the most commonly used are the Löwenstein–Jensen solid-medium method (L-J solid-medium) and the automated liquid-based ratio method, BACTEC MGIT 960. Both methods are essential in the diagnosis of DR-TB and are considered reference standards in microbiology laboratories. However, a major disadvantage of these phenotypic approaches is the time required to obtain results, due to the slow growth rate of *M. tuberculosis*. While the liquid-based MGIT method provides results in a relatively short time (average 7.5 ± 1.8 days), the L-J solid-medium culture requires 28–35 days for complete interpretation, which delays therapeutic decisions [15].

The WHO has approved several genotypic DST methods, including Xpert MTB/RIF and line probe tests (GenoType MTBDR*plus* and MTBDRsl), which have significantly improved the rapid diagnosis of TB and DR-TB. Among them, GenoType MTBDR*plus* is widely used for the detection of rifampicin (RIF) and isoniazid (INH) resistance in clinical specimens and *M. tuberculosis* isolates [16,17].

Resistance of *M. tuberculosis* to antituberculosis drugs represents a major obstacle to the effective control of TB. The mechanisms involved in the emergence of resistance are complex and, although partially elucidated, they can be classified into two broad categories: intrinsic resistance and acquired resistance [18].

Intrinsic resistance is determined by factors that reduce the effectiveness of drugs without the involvement of genetic mutations, such as low cell wall permeability, the activity of efflux pumps, and the peculiarities of bacterial metabolism. In contrast, acquired resistance occurs as a result of mutations in target genes, being the predominant mechanism in *M. tuberculosis* and involving changes that prevent the specific action of drugs [18].

The best-characterized mutations are those involved in resistance to INH and RIF, the two essential first-line drugs. Resistance to INH is mainly caused by mutations in the *katG* gene, which encodes the catalase-peroxidase enzyme responsible for activating the inactive form of isoniazid. Mutations in the promoter region of the *inhA* gene also lead to overexpression of the enzyme *inhA*, essential in the biosynthesis of mycolic acids, reducing the drug’s efficacy [19].

Resistance to RIF is caused, in most cases, by mutations in the *rpoB* gene, which encodes the β subunit of RNA polymerase. These mutations cause amino acid substitutions in the RIF binding region, altering the conformation of the enzyme and reducing or completely blocking its affinity for the antibiotic. Thus, the mechanism of action of RIF is compromised, and the bacillus develops resistance [20].

The most common mutations occur in the so-called RIF resistance region, located between codons 507 and 533, with codons 531, 526, and 516 reporting the strongest associations with resistance. Minimum inhibitory concentration studies have shown that mutations at codons 531 and 526 are correlated with high levels of resistance, while mutations at codons 511, 516, 518, and 522 are associated with low-level forms of resistance [18].

The situation is becoming alarming with the emergence of XDR-TB strains, which are resistant not only to INH and RIF but also to fluoroquinolones and at least one second-line injectable drug, such as kanamycin (KM), amikacin (AMK), or capreomycin (CAP). Fluoroquinolones act by inhibiting DNA gyrase, a key enzyme in bacterial DNA replication; mutations in the gyrA and gyrB genes confer resistance to this class [21]. In turn, mutations in the rrs gene, which encodes the 16S rRNA of the 30S ribosomal subunit, are associated with resistance to injectable aminoglycosides used in the treatment of TB.

Although phenotypic and genotypic methods are complementary in *M. tuberculosis* susceptibility testing, the results obtained can sometimes be discordant, which requires careful interpretation correlated with the clinical and microbiological context.

In this context, our study aims to evaluate the drug resistance profile, the line probe assay (LPA) GenoType MTBDR*plus* molecular method in relation to DST, for the detection of *M. tuberculosis* resistance, and to characterize the mutations of *M. tuberculosis* isolates.

## 2. Results

### 2.1. Description of Patients by Gender, Age Range, Product, and Patient Category According to Resistance/Sensitivity to RIF and INH

Demographic data, such as patient age and gender, product type, and patient category, were recorded and analyzed in relation to resistance/susceptibility to RIF and INH for strains isolated from 792 patients by the L-J solid medium susceptibility testing method. The distribution of patients by gender shows that the majority of patients in this study were male (67.93%). The age of the patients ranged from 4 months to 93 years, with a mean age at diagnosis of 48 years (Table 1).

Of the patients with confirmed TB, 8.83% were diagnosed with MDR-TB, 13.38% had INH monoresistance, and isolated RIF resistance was reported in only 0.37% of patients. Most patients (77.39%) were susceptible to both drugs.

In the case of drug resistance, 5.9% of the total number of female patients presented MDR-TB, and 11.4% were observed to have INH monoresistance. In the case of male patients, most presented with INH monoresistance (14.3%), followed by MDR-TB at 10.2%, and only 0.6% presented with isolated RIF resistance. For the majority age group in this study (40–60 years), 13.9% of patients presented with INH monoresistance, and 10.9% reported resistance to both drugs. The majority of patients (94.32%) were diagnosed with pulmonary TB (*n* = 747), while 5.68% of patients (*n* = 45) presented with extrapulmonary TB. The distribution of cases according to respiratory products revealed that most samples were represented by sputum (98.12%). Among the cases of extrapulmonary TB, more than half were puncture fluids, followed by a few gastric aspirates, cerebrospinal fluid, and purulent lymph node secretions.

In the case of respiratory samples, although most cases were sensitive to the drugs, the highest level of resistance was observed to INH (13.38%), followed by RIF with a percentage of 9.23%. Non-respiratory samples showed multiple or isolated resistance only in limited cases, showing a sensitivity to both drugs of 84.44%. In the case of less common pathological products, such as biological fluids and gastric aspirate, sensitivity to both drugs prevailed, representing 78.26% and 90%, respectively.

Of the total patients, 84.97% were classified as new cases, of which 5.3% showed resistance to both RIF and INH. Relapses represented 10.6%, with a prevalence of MDR-TB of 11.9%. Regarding chronic cases (4.41%), the proportion of MDR-TB was significantly higher, reaching 68.6%.

**Figure 1:**
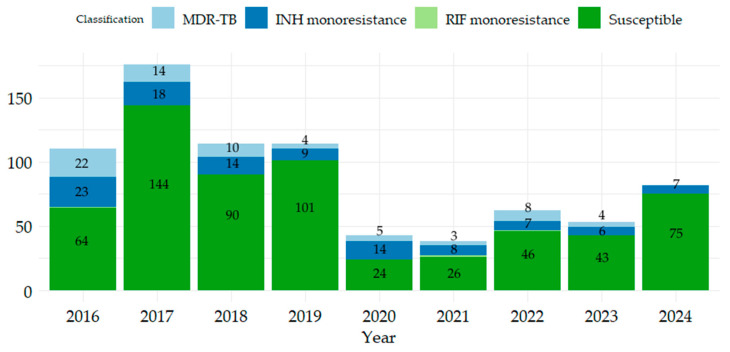
Distribution of TB patients registered in the period 2016–2024, and susceptibility of isolated strains to INH, RIF.

Of the total of 792 patients, the highest number who presented with TB were registered in 2017 (22.22%), and the fewest in 2020 and 2021, with 43 and 38 patients, respectively (Figure 1). The highest number of patients who presented with MDR-TB was in 2016 (20% of the total patients in that year). The highest number of patients with monoresistance to INH was reported in 2016 (20.9% of the total patients in that year). Monoresistance to RIF was observed in three patients: one patient each in 2016, 2021, and 2022. The number of patients susceptible to both drugs ranged from 24 in 2020 to 144 in 2017.

### 2.2. Comparison of Phenotypic, Genotypic Methods

In this study, the performance of the GenoType MTBDR*plus* assay for the identification of RIF and INH resistance in *M. tuberculosis* was evaluated. The GenoType MTBDR*plus* assay was compared with the phenotypic drug susceptibility testing (DST), which was used as the gold standard, on a total of 513 isolates common to both methods. To determine the performance of the GenoType MTBDR*plus*, sensitivity (SN), specificity (SP), accuracy (A), positive predictive value (PPV), and negative predictive value (NPV) were calculated (Table 2).

Compared to the DST method, the GenoType MTBDR*plus* assay demonstrated a sensitivity of 94.74% and a specificity of 99.39% for detecting rifampicin resistance, indicating high performance. For INH, the assay demonstrated a sensitivity of 95.16% and a specificity of 99.56%. The overall accuracy (total proportion of correct results) exceeded 99% for both drugs. In terms of correctly identifying resistant cases, the PPV was higher for INH (96.72%) than for RIF (85.71%).

### 2.3. Analysis of Mutations Associated with Resistance to RIF and INH

Mutations associated with RIF resistance in the *rpoB* gene, as well as those involved in INH resistance, were characterized—namely high-level mutations in the *katG* gene and low-level mutations in the promoter region of the *inhA* gene for 513 patients (Table 3).

Resistance to RIF was explored by identifying mutations in the RRD region of the *rpoB* gene. Of the total of 21 cases detected, the most frequent mutation was MUT3 (S531), and this was predominantly observed in patients in the categories of new cases (1.30%) and relapse cases (42.85%). The MUT2B (H526D) mutation, although rare, was detected in only one case (2.12%) in the recessive cases category. The MUT1 (D516V) mutation was identified in two patients: one new case and one chronic case. The rpoB WT4 (del 518) mutation was observed in four cases, accounting for 0.87% of new cases and 28.57% of chronic cases.

INH resistance was detected in 59 *M. tuberculosis* strains, and mutation analysis showed that the katG 315 mutation was present in 84.74% of the INH-resistant isolates. This was observed in 7.84% of new cases, 17.02% of relapsed cases, and 85.71% of chronic cases. All isolates with mutations in katG 315 hybridized with the katG MUT1 probe, indicating the AGC-ACC substitution, and the katG MUT2 probe showed no hybridization, suggesting the absence of other mutations in codon 315. Mutations in the *inhA* promoter region were identified in seven patients, all being new cases, and *katG/inhA* mutations in two other patients, also new cases. In the case of mutations in the *inhA* promoter region, the distribution was as follows: the inhA MUT2 probe was detected in most cases, and no cases with hybridization to the inhA MUT3A or inhA MUT3B probes were observed.

The majority of strains tested did not exhibit resistance-associated mutations to either RIF (95.91%) or INH (88.50%), indicating phenotypic sensitivity.

### 2.4. Phenotypic Resistance to First-Line and Second-Line Drugs

Of the total 792 strains isolated and explored by the DST method, 66 isolates included both monoresistance to isoniazid and combined resistance to isoniazid and rifampicin. In total, 43.93% of the isolates were classified as MDR-TB (resistant to both RIF and INH).

Regarding first-line drugs, phenotypic resistance was 46.97% for RIF, 84.85% for INH, 48.48% streptomycin (STR), and 30.30% for ethambutol (EMB). A significant aspect is the fact that 22.73% of MDR-TB isolates were resistant to all four first-line drugs, i.e., one-fifth of the isolates.

**Figure 2:**
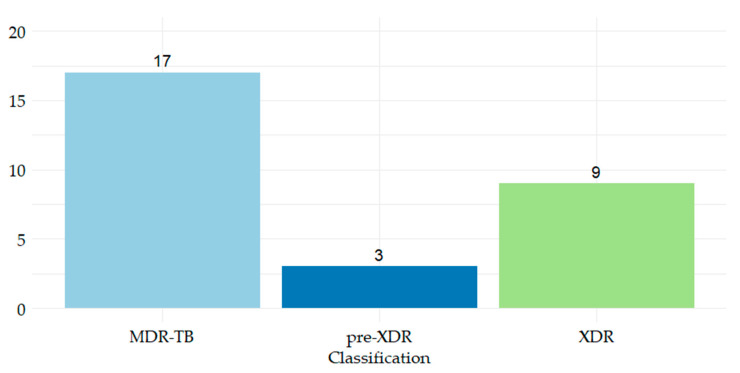
Phenotypic resistance to first and second-line TB drugs and classification of resistance. MDR-TB represents resistance to RIF and INH, pre-XDR represents resistance to RIF and INH and OFL and (LVX or MFX), and XDR represents resistance to RIF and INH and OFL and (KM or ETM or AKM or CAP).

For second-line drugs, regarding MDR-TB isolates, 41.37% showed resistance to fluoroquinolones (Ofloxacin (OFL), levofloxacin (LVX), moxifloxacin (MFX)), and 31.03% were resistant to both fluoroquinolones and injectable aminoglycosides (Figure 2).

Of the 29 MDR-TB patients, 41.37% of cases were classified as pre-XDR TB, of which 91.67% were resistant to OFL, and 8.33% were resistant to both LVX and MFX. The evolution of these patients showed a high rate of progression to XDR-TB, with 75% of pre-XDR cases subsequently developing XDR-TB. Compared to the entire group of MDR-TB patients, 31.03% progressed to XDR tuberculosis.

## 3. Discussion

In recent years, new technologies have been introduced to reduce diagnostic time and improve methods for detecting drug resistance, both at the molecular and phenotypic levels. However, with the increasing use of these tests, discrepancies in the results obtained have been reported, making their evaluation useful [22].

The results of our study highlight the prevalence of MDR-TB by sex, age, and patient category, the results being comparable to other relevant research. According to a 2020 report, out of 358 MDR-TB cases in Romania, 78% were recorded in men, with a median age of 47 years (range: 16–91 years) [12]. Also, a 2023 global study revealed a higher prevalence of MDR-TB in men (9.39 cases per 100,000 population) compared to women (7.95 cases per 100,000 population) [23]. These trends are also supported by recent data from Europe. For example, in Poland, a study analyzing the evolution of MDR/RR-TB cases between 2010 and 2021 reported that 77% of patients were male, and the median age has decreased from 49 to 42 years in recent years, indicating increased incidence among younger adults [24].

Other European research has shown that the age groups with the highest incidence of MDR-TB and XDR-TB are 35–44 and 55–64 years, and the risk is increased among previously treated patients and those born outside the EU [10].

In Romania, the TB incidence rate, although decreasing, remains one of the highest in Europe, highlighting the need for strategies adapted to local epidemiological realities [25]. The distribution of TB cases and drug resistance reflects similar trends to those reported internationally, providing relevant insight into current challenges in the management of this disease. The proportion of 84.97% of new cases observed is consistent with global statistics, with the WHO reporting that the majority of TB cases worldwide in 2023 were new cases [25]. Although chronic cases represent only 4.41%, they are particularly problematic due to the high prevalence of MDR-TB. Our data show that MDR-TB is present in 68.57% of all chronic cases and in 11.90% of all relapsed cases, figures comparable to international reports.

The results of our study revealed a high level of concordance between the methods and GenoType MTBDR*plus* and DST, underlining the applicability of molecular testing in the diagnosis and management of MDR-TB. Specifically, the concordance between GenoType MTBDR*plus* and the phenotypic method was 94.74% for RIF and 95.16% for INH, values comparable to those in the literature. For example, a study from Vietnam reported a sensitivity of 93.1% for RIF and 92.6% for INH, with a specificity of 100% [26]. Similarly, a study from Morocco showed a concordance of 90.8% for RIF and 96.5% for INH [27].

Although our study did not directly compare the GenoType MTBDR*plus* assay to GeneXpert MTB/RIF, literature data suggest that GenoType MTBDR*plus* has superior performance in detecting drug resistance. However, the implementation of LPA tests, such as GenoType MTBDR*plus*, at the point of care remains challenging in resource-limited countries due to the need for advanced laboratory infrastructure and specialized personnel [28]. The genotypic method (Genotype MTBDRplus) provides results within approximately 1–2 days after sample processing, while the phenotypic method using Löwenstein–Jensen medium generally requires 4–8 weeks, depending on the growth rate of the bacilli. This difference has major clinical implications, as the rapid molecular method enables earlier initiation of appropriate therapy, thereby reducing the risk of transmission and disease progression.

Discrepancies between genotypic and phenotypic tests can raise important clinical issues. In our study, we observed discrepancies between the LPA method and the phenotypic method in three isolates (14.3%) for RIF and two isolates (3.3%) for INH. These discrepancies are attributed to the absence of known resistance mutations in isolates identified as resistant by phenotypic testing and the detection of low-level mutations in isolates susceptible to RIF by phenotypic testing [29,30,31].

Hr-TB in the absence of RR-TB was the most common form of resistance in our study, identified in 59 strains. This requires the addition of a fluoroquinolone to the therapeutic regimen, and in the case of low-level mutations in the *inhA* gene, the administration of increased doses of INH may be considered [32]. Mutations in codon 315 of the *katG* gene confer high resistance to INH, precluding the use of this drug [33].

In this study, susceptibility testing using the GenoType MTBDR*plus* method identified mutations in the *katG* gene representing 84.74% of INH-resistant isolates and 9.74% of the total samples analyzed. This mutation is associated with a high risk of treatment failure if INH is continued, even at high doses, and thus INH should typically be excluded from the regimen [34]. Mutations in *inhA* were detected in 11.86% of INH-resistant isolates and 1.36% of the entire batch. Mutations in the *inhA* promoter region (particularly C-15T) are linked to low-level INH resistance by upregulating InhA expression. In such cases, high-dose INH (10–15 mg/kg/day) may retain partial efficacy, which is reflected in WHO treatment guidelines [6]. However, these mutations are also associated with cross-resistance to ethionamide and prothionamide, drugs with overlapping targets, thereby limiting alternative treatment options [35]. These results highlight the distinct relationship between *katG* and *inhA* mutations and emphasize the importance of genotypic testing for optimizing MDR-TB treatment. Data obtained are consistent with other studies, which indicate *katG* as the main genetic factor associated with INH resistance, usually correlated with high-level resistance. By contrast, mutations in the *inhA* gene promoter are generally associated with low-level resistance [33].

Resistance to RIF, an essential drug in the treatment of tuberculosis, is determined by mutations in the *rpoB* gene, which encodes the β subunit of RNA polymerase. These mutations induce conformational changes in the RIF binding site, reducing its affinity for the drug and promoting resistance. Studies have shown that the most common mutations are in the region between codons 507–533, with significant clinical impact. Of these, mutations at codons 531 and 526 are associated with high levels of resistance, while those at codons 511, 516, 518, and 522 are linked to lower levels of resistance [18].

In the present study, the rpoB Mut3 (S531L) mutation was frequently identified, being present in both RIF-monoresistant and MDR-TB isolates. This result is similar to data reported in other studies, where the S531L mutation is predominant. For example, in a study from India, this mutation was detected in 72% of the MDR-TB strains analyzed, and the frequency observed in our study is comparable to results from other regions [36,37]. Regarding rpoB mutations, our findings are consistent with previous data showing that S531L is the most prevalent mutation, strongly associated with high-level rifampicin (RIF) resistance and poor treatment outcomes [38]. Other mutations, such as D516V, have been correlated with low-level RIF resistance, and in some cases, preserved susceptibility to rifabutin has been documented [39]. Such distinctions may have therapeutic relevance, particularly in individualized treatment regimens where rifabutin could be considered if confirmed by additional phenotypic or MIC testing.

The variability in the distribution of these mutations suggests the existence of geographical differences in RIF resistance, which underlines the importance of continuous monitoring of the genetic profile of *M. tuberculosis* in diverse populations. The identification of the S531L mutation in both monoresistant and MDR-TB strains confirms its major role as a molecular marker of RIF resistance [30].

Regarding the H526D (MUT2B) and D516V (MUT1) mutations, the frequencies observed in our study were 0.19% and 0.38%, respectively. These values are comparable to those previously reported, where the H526D mutation was identified in 4% of MDR-TB strains and D516V in 2% [37]. Studies in other regions, such as Canada, have also reported that the S531L, H526Y, and H526D mutations are associated with high levels of RIF resistance [40]. In our study, we identified three cases of RIF monoresistance, supporting the observation that although RIF resistance is frequently associated with MDR-TB, isolated cases of monoresistance may also exist. This identification is essential, as RIF monoresistant strains are associated with a lower rate of treatment success and pose an increased risk of progression to MDR-TB in the absence of appropriate treatment. According to the WHO, globally, approximately 1.1% of TB patients present RIF monoresistance, but these values vary significantly by region, reaching as high as 20% in some areas. RIF monoresistance is also more frequently reported in HIV-positive patients, which highlights the need for HIV-specific treatment strategies [41].

Although molecular tests, such as GenoType MTBDR*plus*, are rapid and accurate methods for detecting rpoB mutations, they have important limitations. False negative results for some resistance-associated mutations, such as rpoB I491F, are not detected by WHO-approved molecular tests, which may lead to underdiagnosis. In addition, heteroresistant strains may be overlooked due to low mutation frequencies [33].

Although heteroresistance in *M. tuberculosis* has been widely reported, its detection in clinical samples is challenging, especially when using currently available molecular methods. These tests may miss resistant subpopulations present in small proportions (below 1%), a threshold generally detectable only by traditional culture-based phenotypic drug susceptibility testing methods. Therefore, molecular methods should be used in combination to assess drug susceptibility of *M. tuberculosis* strains [42].

Genotypic tests can generate false-positive results in detecting drug resistance, especially when they misinterpret mutations that do not influence treatment sensitivity. For example, the rpoB T427A mutation, considered a neutral mutation, may be erroneously classified as associated with rifampicin resistance, which may lead to inappropriate therapeutic decisions [33].

Additionally, technical limitations of older versions of molecular tests can affect diagnostic accuracy. For example, the GenoType MTBDR*plus* test version 2 (2011) did not detect the borderline L452P mutation, which could lead to false negative results. This deficiency was only corrected in later versions of the test, released in 2014 and 2019 [43].

Our results highlight a significant prevalence of pre-XDR-TB and XDR-TB among MDR-TB patients, confirming trends reported in other international studies. The rapid progression from pre-XDR to XDR-TB underscores the severity and complexity of these infections, which revealed the need for effective therapeutic regimens and improved access to DST. These findings are consistent with the literature, which indicates that the presence of fluoroquinolone resistance, especially to OFL and LVX, increases the risk of progression to XDR-TB and limits therapeutic options. Although the WHO recommends the use of new-generation drugs bedaquiline (BDQ), delamanid (DLM), linezolid (LZD), and clofazimine (CFZ), the lack of DST testing for these agents in many regions, including Romania, may compromise personalized treatment strategies [44].

Our study has several limitations that should be considered when interpreting the results. First, we did not use the minimum inhibitory concentration determination, a valuable method for confirming susceptibility test results and adjusting therapeutic regimens, especially in cases of suspected borderline resistance. The absence of this method may limit the accuracy of assessing the true level of resistance.

Also, the lack of DNA sequencing to clarify discordant results between genotypic and phenotypic tests, as well as the non-use of revised WHO breakpoints for rifampicin in low-level resistance forms, represent important methodological limitations [18].

In addition, the correlation of microbiological data with the clinical outcome of patients was not performed, which restricts the assessment of the impact of resistance on treatment response. Data collection from a single center implies a geographical limitation, which may reduce the applicability of the results at the national or international level, and a low sample size.

Last but not least, the use of the L-J medium proportions method, although standardized, is limited by the long working time and the low sensitivity in detecting low-level resistance, aspects that may influence diagnostic accuracy.

The integration of advanced molecular technologies in reference laboratories, although expensive, is essential for improving diagnosis [29]. Thus, our data support the need to expand access to molecular and phenotypic resistance testing, as well as optimize therapeutic protocols for patients with MDR/XDR-TB, in line with international recommendations.

To better correlate genetic mutations with phenotypic resistance, future studies should combine DST with whole genome sequencing (WGS), thus ensuring a personalized and effective therapeutic approach [18].

## 4. Materials and Methods

### 4.1. Data Collection

Our retrospective study, conducted between January 2016 and August 2024, included patients with pulmonary and extrapulmonary TB, microbiologically confirmed by culture on L-J solid medium and molecular tests, admitted to the Bihor County Emergency Clinical Hospital. All patients with confirmed tuberculosis were included. Confirmation of the diagnosis was based on microscopic examination of Acid-Fast Bacilli-stained smears and culture on L-J medium. Inclusion criteria assumed identification of *M. tuberculosis* by culture, and patients without cultural confirmation were excluded from the study. All patients are registered in the National Tuberculosis Registry.

Data were collected from the “Form for requesting/reporting bacteriological examination for TB”, according to the requirements of the National Tuberculosis Program, and from the TB Microbiology Laboratory Registry. The data collected included socio-demographic characteristics: age, gender, case category (new case, relapse case, chronic case), clinical characteristics, chest X-ray examination results, and other relevant data regarding the clinical condition. The results of bacteriological investigations, including smear, culture, and drug sensitivity test results, were useful for case classification. The following categories of cases were defined:

- New case: Patients who have never been treated for TB or who have received anti-TB drugs for less than a month.
- Relapse case: Patients previously declared “cured” or “treatment completed” who develop active TB again, bacteriologically confirmed by positive culture for *M. tuberculosis*. Relapses are considered if the recurrence of the disease is diagnosed at least 4 months after completion of previous treatment.
- Chronic case: Patients who have completed a full course of treatment, including relapse, but who continue to be positive for *M. tuberculosis* on bacteriological culture. Persistent disease is confirmed when the culture remains positive at least 12 months after the initiation of the first treatment, indicating failure of bacteriological cure.

### 4.2. Drug Susceptibility Testing (Laboratory Procedure)

*M. tuberculosis* isolates were tested for drug susceptibility using: the L-J solid medium susceptibility testing method, GenoType MTBDR*plus* for the detection of genetic mutations associated with resistance to INH and RIF, and DST for first-line drugs according to the latest technical recommendations [45]. Monoresistant Hr-TB and MDR-TB strains were subsequently tested for second-line drugs at the National Reference Laboratory, using the L-J solid medium proportion method.

#### 4.2.1. Löwenstein–Jensen Solid Media Sensitivity Test Method

The traditional phenotypic method was used for the isolation and cultivation of M. tuberculosis on solid L-J medium. Antituberculosis drug susceptibility testing was performed using absolute concentration and ratio methods, applied to both first-line drugs (RIF, INH, EMB, STR) and second-line drugs (OFL, AMK, KM, CAP, ethionamide (ETH), LVX, MFX). For each drug, the critical concentrations recommended by the WHO were used, as follows: INH (0.2 mg/L), RIF (40 mg/L), EMB (2 mg/L), STR (4 mg/L), LVX, MFX, OFL (2 mg/L), AMK, KM (30 mg/L), and CAP (40 mg/L). These critical concentrations were adapted in accordance with international conventions, being defined as the lowest concentrations that inhibit the growth of 99% of phenotypically wild-type M. tuberculosis strains (90% for pyrazinamide).

To assess resistance to second-line drugs, the method of proportions was used, considered the gold standard in determining antibiotic susceptibility. This involves comparing bacterial growth on media containing anti-TB agents with control media without antibiotics. The procedure included the preparation of two sets of media: one with the critical concentration of the drug and one control without the antibiotic. Inoculation was performed as follows: the anti-TB agent medium was inoculated with a 10^−2^ dilution of a suspension adjusted to the McFarland 1 standard, and the control medium with a 10^−4^ dilution. The results were interpreted by comparing the number of colonies and calculating the bacterial growth ratio, expressed as a percentage. To ensure the quality of the tests, each batch was checked by internal control, using *M. tuberculosis* H37Rv (ATCC 27294) as the standardized reference strain for drug susceptibility.

#### 4.2.2. GenoType MTBDRplus

Molecular diagnosis of anti-TB drug resistance was performed using the GenoType MTBDR*plus* line probe assay (version 2.0, HAIN Lifescience, Nehren, Germany), a method that combines PCR technology with reverse hybridization to enable rapid detection of genetic mutations associated with drug resistance. The assay is designed to identify the *M. tuberculosis* complex and to detect mutations conferring resistance to RIF (by analysis of the *rpoB* gene) and INH (by analysis of the *katG* and *inhA* genes). The procedure included three essential steps: DNA extraction from positive *M. tuberculosis* cultures, DNA amplification with biotinylated primers (AM-A and AM-B), and reverse hybridization on membranes to identify specific mutations. Interpretation of the results was performed in accordance with WHO guidelines, and all testing was performed by trained personnel, following standard protocols and the manufacturer’s instructions.

To ensure the reliability of the test, quality control included the use of a negative control, represented by PCR water, to detect possible contamination, and a positive control, the reference strain *M. tuberculosis* ATCC 25177 (H37Ra), a strain susceptible to first-line drugs. The internal quality control system of the test included five control zones on the hybridization strip: the conjugate control zone (CC), which verifies the efficient binding of the conjugate; the amplification control zone (AC), which confirms the efficiency of the PCR reaction; and three control zones for the *rpoB, katG* and *inhA* genes, which ensure the sensitivity and specificity of the reaction for the genes analyzed.

Minimum inhibitory concentration (MIC) testing and whole genome sequencing (WGS) were not included in this study due to technical and logistical constraints. During the study period (2016–2023), these methods were not routinely available in our laboratory, being limited to specialized national reference centers. Furthermore, the primary aim of our research was to compare phenotypic and genotypic drug susceptibility testing methods that are widely used in routine practice and endorsed by the WHO—specifically, the Löwenstein–Jensen proportion method and the GenoType MTBDRplus assay.

### 4.3. Data Analysis

Microsoft Excel and open-source R software were used for data analysis. Results obtained by molecular and phenotypic methods were initially analyzed for the RIF and INH-resistant population. These results were compared to assess the degree of concordance and discordance between the two methods: DST and genotypic testing. For each method, the sensitivity (SE), specificity (SP), accuracy (A), and positive and negative predictive values (PPV and NPV) were evaluated. In addition, the ability of genotypic methods to identify specific genetic mutations associated with resistance to anti-TB drugs was analyzed.

### 4.4. Ethical Considerations

This study was approved by the Ethics Committee of the Bihor County Emergency Clinical Hospital (11325/05.04.2024). All methods used in this study were performed in accordance with relevant guidelines and regulations. Each participant signed informed consent before undergoing examination.

## 5. Conclusions

The results of our study confirm the effectiveness of the GenoType MTBDR*plus* method as a rapid and accurate tool for identifying resistance to first-line antituberculosis drugs. The high level of concordance between phenotypic and molecular testing supports their integration in the diagnosis of MDR-TB, although the observed discrepancies highlight the need for complementary methods to validate the results.

Our study highlights a significant prevalence of MDR-TB and XDR-TB, confirming international trends and the negative impact of fluoroquinolone resistance on disease progression.

The integration of molecular and phenotypic methods into routine diagnostics, along with improved access to DST for next-generation drugs, is essential for improving the prognosis of patients with drug-resistant TB and limiting the epidemiological impact of the disease.

## Figures and Tables

**Table 1:** Distribution of patients by gender, age range, sample, and patient category according to resistance/sensitivity to RIF and INF.

Category		RIF-R and INH-R	RIF-R and INH-S	RIF-S and INH-R	RMP-S and INH-S	Category Subtotal
**Gender**	F	15	-	29	210	254
	M	55	3	77	403	538
**Age**	(0,20]	2	-	5	40	47
	(20,40]	24	1	26	155	206
	(40,60]	36	1	46	248	331
	(60,80]	8	1	25	141	175
	(80,100]	-	-	4	29	33
**Sample**	Respiratory	69	3	100	575	747
	Non-respiratory	1	-	6	38	45
**Patient category**	New case	36	2	76	559	673
	Recurrence case	10	1	21	52	84
	Chronic case	24	-	9	2	35
**Total**		70	3	106	613	792

R = resistant, S = susceptible; respiratory samples include sputum, induced sputum, tracheal aspirate, bronchial aspirate; non-respiratory samples include gastric aspirate, biological fluids, pus.

**Table 2:** GenoType MTBDR*plus* performance compared to phenotypic drug susceptibility testing (DST) as reference method.

	DST-RIF			SE (95% CI)	SP (95% CI)	A (95% CI)	PPV (95% CI)	NPV (95% CI)
**GenoType MTBD*plus***	R	S	Total	94.74% (0.7397, 0.9987)	99.39% (0.9824, 0.9987)	99.22% (0.9802, 0.9979)	85.71% (0.6366, 0.9695)	99.80% (0.9887, 0.9999)
detected	18	3	21					
undetected	1	491	492					
Total	19	494	513					
	**DST-INH**							
**GenoType MTBD*plus***	R	S	Total	95.16% (0.8650, 0.9899)	99.56% (0.9841, 0.9995)	99.03% (0.9774, 0.9968)	96.72% (0.8865, 0.9960)	99.34% (0.9807, 0.9986)
detected	59	2	61					
undetected	3	449	452					
Total	62	451	513					

R = resistant, S = susceptible, SE = sensitivity, SP = specificity, A = accuracy, PPV = positive predictive value, NPV = negative predictive value.

**Table 3:** Association of mutations according to eligible cases.

Gene/Mutation		New Cases (%)	Recurrent Cases (%)	Chronic Cases (%)
*rpoB*	rpoBMUT1 (D516V)	1 (0.21%)	-	1 (14.28%)
	rpoBMUT2B (H526D)	-	1 (2.12%)	-
	rpoBMUT3 (S531L)	6 (1.30%)	1 (2.12%)	3 (42.85%)
	rpoBWT4 (del 518)	4 (0.87%)	-	2 (28.57%)
	rpoB/WT4	-	1 (2.12%)	-
*katG*	katG (Ser315Thr)	36 (7.84%)	8 (17.02%)	6 (85.71%)
*inh A*		7 (1.52%)	-	-
*katG*/*inhA*		2 (0.43%)	-	-
Total		459	47	7

## Data Availability

The original contributions presented in this study are included in the article. Further inquiries can be directed to the corresponding author.

## Abbreviations

The following abbreviations are used in this manuscript:

BDQ	Bedaquiline
CAP	Capreomycin
CFZ	Clofazimine
DLM	Delamanid
DR-TB	Drug-resistant tuberculosis
DST	Drug susceptibility tests
EMB	Ethambutol
ETH	Ethionamide
GenoType MTBDR*plus*	LPA GenoType MTBDRplus molecular method
Hr-TB	Isoniazid-resistant TB
INH	Isoniazid
KM	Kanamycin
L-J solid-medium	Löwenstein–Jensen solid-medium method ()
LPA	Line probe assay
LVX	Levofloxacin
LZD	Linezolid
MDR-TB	Multidrug-resistant tuberculosis
MFX	Moxifloxacin
MGIT	BACTEC MGIT 960
OFL	Ofloxacin
PCR	Polymerase Chain Reaction
pre-XDR-TB	Pre-extensive drug-resistant TB
RDR	Resistance Determining Region
RIF	Rifampicin
RR-TB	Rifampicin-resistant tuberculosis
STR	Streptomycin
TB	Tuberculosis
WGS	Whole genome sequencing
WHO	World Health Organization
XDR-TB	Extensively drug-resistant tuberculosis
